# Schmallenberg virus: experimental infection in goats and bucks

**DOI:** 10.1186/s12917-015-0516-4

**Published:** 2015-08-22

**Authors:** E. Laloy, M. Riou, C. Barc, G. Belbis, E. Bréard, S. Breton, N. Cordonnier, D. Crochet, R. Delaunay, J. Moreau, N. Pozzi, M. Raimbourg, P. Sarradin, S. Trapp, C. Viarouge, S. Zientara, C. Ponsart

**Affiliations:** Université Paris-Est, Ecole Nationale Vétérinaire d’Alfort, Unité d’anatomie pathologique, 7 avenue du Général de Gaulle, 94704 Maisons-Alfort, France; INRA Centre Val de Loire, UE-1277 Plateforme d’Infectiologie Expérimentale, secteur 3, route de Crotelles, 37380 Nouzilly, France; Université Paris-Est, Ecole Nationale Vétérinaire d’Alfort, Unité de pathologie des animaux de production, 7 avenue du Général de Gaulle, 94704 Maisons-Alfort, France; ANSES, UMR 1161 Virologie ANSES-INRA-ENVA, 23 avenue du Général de Gaulle, 94704 Maisons-Alfort, France; INRA Centre Val de Loire, UMR 1282 Infectiologie et Santé Publique, 37380 Nouzilly, France; Université François Rabelais de Tours, UMR 1282 Infectiologie et Santé Publique, 37000 Tours, France; LNCR, Laboratoire national de contrôle des reproducteurs, 13 rue Jouët, 94703 Maisons-Alfort, France

## Abstract

**Background:**

Schmallenberg virus (SBV) is an emerging *Orthobunyavirus* of ruminant livestock species currently circulating in Europe. SBV causes a subclinical or mild disease in adult animals but vertical transmission to pregnant dams may lead to severe malformations in the offspring. Data on the onset of clinical signs, viremia and seroconversion in experimentally infected adult animals are available for cattle and sheep but are still lacking for goats.

For a better understanding of the pathogenesis of SBV infection in adult ruminants, we carried out experimental infections in adult goats. Our specific objectives were: (i) to record clinical signs, viremia and seroconversion; (ii) to monitor viral excretion in the semen of infected bucks; (iii) to determine in which tissues SBV replication took place and virus-induced lesions developed.

**Results:**

Four goats and two bucks were inoculated with SBV. Virus inoculation was followed by a short viremic phase lasting 3 to 4 days and a seroconversion occurring between days 7 and 14 pi in all animals. The inoculated goats did not display any clinical signs, gross lesions or histological lesions. Viral genomic RNA was found in one ovary but could not be detected in other organs. SBV RNA was not found in the semen samples collected from two inoculated bucks.

**Conclusions:**

In the four goats and two bucks, the kinetics of viremia and seroconversion appeared similar to those previously described for sheep and cattle. Our limited set of data provides no evidence of viral excretion in buck semen.

## Background

In the late summer/autumn 2011, a disease outbreak with diarrhea, drop of milk production, and fever was reported in adult cattle in Western Europe. These symptoms could not be attributed to any known infectious agent. Metagenomic analyses on blood samples from affected animals in Germany led to the identification of a new *Orthobunyavirus* that was named the Schmallenberg virus (SBV) [1]. This emerging virus was later found to induce teratogenesis in pregnant cattle, sheep, and goats leading to typical malformations in the offspring [2].

Experimental infections of adult sheep and cattle with SBV resulted in subclinical infections with a short viremic phase. Seroconversion in the infected animals occurred about two weeks post inoculation (pi) [1, 3–5]. To our knowledge, no report on the pathogenesis of experimental SBV infections in adult goats has been published.

SBV is transmitted by biting midges (*Culicoides* spp.). The possibility of sexual transmission between ruminants has not yet been elucidated [2]. Infectious SBV has been detected in bovine semen samples from the field [6–8] and SBV RNA could be detected in semen from experimentally infected bulls [9]. Whether SBV can be excreted in buck semen is still unknown.

In this study, we carried out experimental infections of SBV in adult goats. Our specific objectives were: (i) to record the development of clinical signs, viremia and seroconversion in goats; (ii) to monitor the excretion of SBV in buck semen after inoculation; (iii) to determine in which tissues SBV replication took place and virus-induced lesions developed in adult bucks and non-gravid goats, with special emphasis on the genital tract.

## Methods

All experiments were conducted in accordance with the guidelines of the Council European Directive (2010/63/UE). All experimental procedures were approved by the ethical review board of the Val de Loire (CEEA VdL, committee number n°19, number 2012-02-11).

### Experimental design

Five adult Alpine goats, one adult Saanen buck and one adult Alpine buck were purchased from local breeders (INRA Center, Bourges, France) and were housed in the Biosafety Level 3 and insect-proof animal facilities of the National Institute of Agricultural Research (INRA), Research Loire Valley Center (PFIE, Nouzilly, France). All purchased animals were SBV-negative as determined by ELISA and RT-qPCR.

Two goats (designated A and B) were inoculated subcutaneously on day 0 with 1 mL of SBV-containing bovine serum kindly provided by the Friedrich-Loeffler-Institut (FLI), Germany [3]. Two goats (designated C and D) were inoculated on day 0 with 1 mL of SBV-containing ovine whole blood collected at the PFIE during a previous experimental infection trial [5]. One goat from each group was killed at day 7 pi and the remaining goats were killed at day 14 pi. The two bucks (designated E and F) were inoculated subcutaneously at day 0 with 1 mL of the FLI serum and killed at day 28 pi. One goat (designated G) was inoculated subcutaneously on day 0 with 1 mL of sterile saline solution and served as an in-contact negative control until it was killed at day 28 pi.

During the course of the trial, all animals were monitored twice daily, and body temperatures were recorded by telemetric measurement with rumen temperature sensors (Small Bolus®, Médria, Châteaubourg–France). After inoculation, whole blood and serum samples were collected daily during the first week and then at days 14 and 28 pi. Buck semen was collected at day 0 and then twice a week. At necropsy, all the organs were macroscopically evaluated and a panel of tissue samples was collected for histopathology and RT-qPCR (spleen, prescapular lymph node, skeletal muscle, aorta, liver, kidney, lung, small intestine, brain, skin, ovary, oviduct, uterus, testis, and epididymis).

### Real-time PCR

Ovaries were dissected and follicular fluid, cumulus cells, oocytes and interstitial tissue were separated from each other prior to total RNA extraction. RNA from blood and tissue samples was extracted using the LSI MagVet™ Universal Isolation kit (Life Technologies SAS, Saint-Aubin, France) and King Fisher magnetic particle processor (Thermo Scientific™, Illkirch, France) according to the manufacturers’ instructions. RNA from semen samples was extracted with Trizol ® LS Reagent [6].

The samples were then tested for the presence of SBV RNA by RT-qPCR as previously described [10]. Quantification cycle (Cq) threshold value was 40, with higher values regarded as negative.

### Serology

Serum samples were submitted to SBV specific ELISA testing (ID Screen Schmallenberg virus Indirect®, monocupule, IDvet) and virus neutralization test (VNT) [11].

### Histopathological examination

After fixation in 10 % buffered formalin, tissues were routinely processed, sliced at 4 μm, stained with Hematoxylin-Eosin-Saffron (HES) and examined by light microscopy.

## Results

### Clinical and post-mortem observations

The goats and bucks did not exhibit any clinical signs. No fever peak was detected in any of the animals. No significant gross lesion was found at necropsy.

### Real-time PCR

**Fig. 1:**
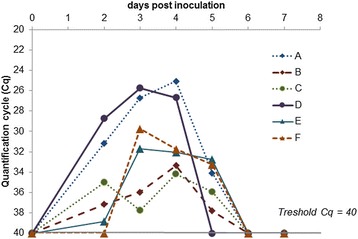
Detection of SBV RNA in blood by RT-qPCR after SBV inoculation in goats and bucks. A, B: goats inoculated with SBV-containing bovine serum; C, D: goats inoculated with SBV-containing ovine blood; E, F: bucks inoculated with SBV-containing bovine serum

SBV RNA was detected in the blood of all inoculated animals for 3 to 4 days, starting between day 1 and 3 pi (Fig. 1). From day 6 pi, SBV RNA became undetectable. Cq values during RNAemia in all animals ranged from 25 to 39. The intensity of RNAemia differed in 2 of the 4 inoculated goats, with goats A and D showing a maximal Cq value of about 25 while goats B and C showed a maximal Cq value of about 35, independently of the inoculum (ovine EDTA blood or bovine serum). All sampled tissues scored negative for SBV by RT-qPCR in bucks and goats, except for one ovary in goat C (interstitial ovarian tissue with a non-normalized Cq value of 34). The semen from the bucks remained negative for SBV from day 0 until the end of the trial, as determined by RT-qPCR.

### Serology

**Fig. 2:**
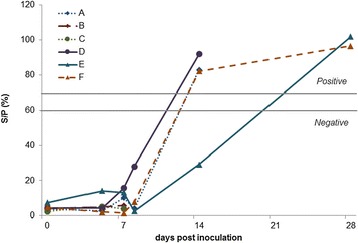
Detection of SBV specific antibodies by ELISA after SBV inoculation in goats and bucks. A, B: goats inoculated with SBV-containing bovine serum; C, D: goats inoculated with SBV-containing ovine blood; E, F: bucks inoculated with SBV-containing bovine serum. S/P < 60 %: negative; S/P > 70 %: positive and S/P between 60 % and 70 %: doubtful

The ELISA results for inoculated animals are shown in Fig. 2. Goats B and C, killed at day 7 pi, remained seronegative. Antibodies to SBV were detected in goats A and D at 14 pi by ELISA and at day 9 pi by VNT (titers: 128 and 96 respectively). Antibodies to SBV were detected in buck E at day 28 pi by ELISA and at day 14 pi by VNT (titer: 64). Buck F was found seropositive at day 14 pi by ELISA and VNT (titer: 96). The mock-inoculated goat remained seronegative until day 28 pi (as determined by ELISA).

### Histopathological examination

No significant lesions was found in any of the animals. Rare coccidian parasites were seen in the intestine from goats B, C and G.

## Discussion

Our results show that, in goats and bucks, inoculation of SBV is followed by a short viremic phase lasting 3 to 4 days followed by seroconversion between day 7 and day 14 pi. Importantly, these clear signs of successful experimental infection were not accompanied by any clinical sign, including fever.

These observations are in accordance with data from experimental infection studies with sheep and cattle inoculated subcutaneously with infectious serum. In sheep, RNAemia was detected a few days after infection and lasted for 3 to 7 days, while SBV-specific antibodies appeared between 7 and 9 days pi (detection by VNT) [12] or between 10 and 14 days pi (detection by ELISA) [5]. Data from this experiment in goats show a trend towards a higher sensitivity of VNT compared to ELISA at the beginning of the seroconversion. Poskin et al. [12] had similar observations in sheep and suggested that this difference could be due to the ability of VNT to detect both IgM and IgG, while the ELISA can only detect IgG. Following experimental infection, sheep did not show any clinical sign [12] or almost no sign, with diarrhea being reported in one case [5]. In cattle, RNAemia could be detected for less than a week after inoculation [1, 3, 4] with detection by ELISA of SBV-specific antibodies two weeks pi [3, 4]. Clinical signs were absent [3, 4] or limited to fever or diarrhea [1].

In cattle and sheep, after experimental inoculation, SBV genomic RNA was most consistently found in lymphoid organs, i.e. spleen and lymph nodes, especially the mesenteric lymph nodes [3–5, 9, 12]. This was not the case in any of the four goats and two bucks inoculated in the present study; however we did not collect the mesenteric lymph nodes. The only organ where SBV genome was found was one ovary of a single goat. Interestingly, the SBV genome had already been found in the ovary of one experimentally infected sheep [5], but the significance of this finding remains unknown. No significant gross lesions or histological lesions were found in our study. Similarly, experimental infections of cattle or sheep did not result in gross lesions at necropsy [3–5, 12]. So far, in experimentally infected adult ruminants, the presence of SBV genome in a given organ has not been reported to be associated with any lesion in this organ.

No SBV genome was found between day 0 and day 28 pi (end of the trial) in the semen samples obtained from the two inoculated bucks. This result differs from the observations made after experimental infection of two bulls with SBV-containing cell culture supernatants [9]. Viral genome could be detected in the semen of both bulls for the first week following inoculation and, in the case of one bull, as late as at day 19 pi (the trial ended at day 25 pi). SBV excretion in bovine semen has also been reported from the field [6–8] but, to our knowledge, this has never been reported for bucks. However, the small size of our inoculated group precludes a conclusion regarding the excretion of SBV in caprine semen and the risk of sexual transmission.

The data from this study match those from reports of natural infection by SBV in domestic ruminants: clinical signs of infection are either mild or absent in adults. The most important effects of SBV infection are malformations in the offspring due to vertical transmission in pregnant dams [2]. A study of the impact of SBV in French domestic ruminants showed that only 2 % of the kids born in goat herds with congenital SBV cases showed malformations [13]. The same study reported a potential effect of SBV infection in the early stages of pregnancy, based on reports of repeated estrus or early embryonic loss, especially in small ruminants [13]. The effects of SBV infection in pregnant goats, however, remain to be elucidated.

## Conclusions

Following experimental infection in goats with SBV, the kinetics of viremia and seroconversion were found to be similar to earlier reported kinetics in sheep and cattle. No clinical signs were associated to infection, in agreement with reports from the field. SBV RNA was found in one ovary but not in other organs. The SBV genome was not found in semen from the two inoculated bucks but this limited set of data does not exclude the risk of viral transmission by artificial insemination or natural service.

## Abbreviations

Cq: Quantification cycle
FLI: Friedrich-Loeffler-Institut
HES: Hematoxylin-eosin-saffron
PFIE: Plate-Forme d’Infectiologie Expérimentale
pi: Post inoculation
SBV: Schmallenberg virus
VNT: Virus neutralization test
